# A retrospective observational study of low-level viraemia and its immunological and virological significance: which outcome to expect

**DOI:** 10.7448/IAS.17.4.19668

**Published:** 2014-11-02

**Authors:** Joana Silva, Karen Pereira, Joao Rijo, Teresa Alberto, Joaquim Cabanas, Perpetua Gomes, Helena Farinha, Kamal Mansinho

**Affiliations:** 1 Infectious Diseases, Hospital Egas Moniz – CHLO, E.P.E., Lisboa, Portugal; 2 Pharmaceutical Department, Hospital Egas Moniz – CHLO, E.P.E., Lisboa, Portugal; 3 Microbiology and Molecular Biology Laboratory, Hospital Egas Moniz – CHLO, E.P.E., Lisboa, Portugal

## Abstract

**Introduction:**

Low-level viraemia (LLV) is observed in some patients with HIV-1 infection on stable antiretroviral therapy (ART). The significance of these findings remains controversial as it conflicts with traditional optimal clinic outcome. This study aims to evaluate the effect of LLV on the establishment of virological failure (VF) and immune deterioration.

**Methods:**

Retrospective observational study of a cohort of HIV-1 infected patients of an Infectious Diseases Clinic, who presented an HIV-1 viral load of 20 to 200 cp/mL, during the year 2012. Patients who were not on ART or non-adherent in the previous 6 months were excluded. Compliance was quantified by clinical and pharmaceutical records. Adherence was defined as ≥95% compliance rate. Demographic, clinical, immunological and therapeutic data were collected from clinical records. LLV was defined as a range of 20–200 cp/mL and stratified as transient (T-LLV): only one measurement, persistent (P-LLV): 2 consecutive measurements with an interval ≥3 months and recurrent (R-LLV): ≥1 T-LLV during an 18-month follow-up. Statistical analysis was performed with Microsoft Office^®^ – Excel 2012. Kolmogorov–Smirnov test, *t*-test and chi-square test were performed for a significant p value <0.05.

**Results:**

During 2012, 2161 HIV-1 infected patients were evaluated at our Clinic, 93% of which were on ART. LLV was documented in 378 (19%), adherence was verified in 151 (52%). The analysis of this cohort (n=151) revealed: 77 (51%) T-LLV, 13 (8.6%) R-LLV and 61 (40%) P-LLV. Mean viral load was 46 cp/mL. Mean TCD4 count was 665 cells/µL with a variation of +63 cells/µL during the study period. There was no VF documented. ART regimens were switched in 16 (11%) patients. Gastrointestinal disturbance was found in 13 (9%). Analysis showed no statistical differences between the analyzed variables (CD4 variation, time of diagnosis and treatment, duration of LLV persistence (less than or more than one year), number of ART regimens, ART regimen and type of NRTI backbone) for all groups (T-LLV, R-LLV, P-LLV), except for mean viral load that showed significant superiority in the T-LLV(38 cp/mL) and R-LLV(36 cp/mL) vs P-LLV(58 cp/mL) (p=0.01 and p<0.01, respectively).

**Conclusions:**

The absence of significant differences in immunological and virological outcomes in this cohort and the absence of VF in all groups, suggests a scarce impact of LLV in patient's prognosis. Prospective studies, with longer follow-up could bring more accurate information.

